# Small-molecule inhibitors of proteasome increase CjCas9 protein stability

**DOI:** 10.1371/journal.pone.0280353

**Published:** 2023-01-19

**Authors:** Pouiré Yaméogo, Nathalie Majeau, Cedric Happi Mbakam, Jacques P. Tremblay

**Affiliations:** 1 Centre de Recherche du CHU de Québec-Université Laval, Québec City, QC, Canada; 2 Département de Médecine Moléculaire, Université Laval, Québec City, QC, Canada; University of South Florida, UNITED STATES

## Abstract

The small size of CjCas9 can make easier its vectorization for *in vivo* gene therapy. However, compared to the SpCas9, the CjCas9 is, in general, less efficient to generate indels in target genes. The factors that affect its efficacity are not yet determined. We observed that the CjCas9 protein expressed in HEK293T cells after transfection of this transgene under a CMV promoter was much lower than the SpCas9 protein in the same conditions. We thus evaluated the effect of proteasome inhibitors on CjCas9 protein stability and its efficiency on *FXN* gene editing. Western blotting showed that the addition of MG132 or bortezomib, significantly increased CjCas9 protein levels in HEK293T and HeLa cells. Moreover, bortezomib increased the level of CjCas9 protein expressed under promoters weaker than CMV such as CBH or EFS but which are specific for certain tissues. Finally, ddPCR quantification showed that bortezomib treatment enhanced CjCas9 efficiency to delete GAA repeat region of *FXN* gene in HEK293T cells. The improvement of CjCas9 protein stability would facilitate its used in CRISPR/Cas system.

## Introduction

CRISPR/Cas9 is an impressive tool for genome editing [1]. Cas9 proteins from different bacterial species have been isolated, each has a different size and a distinct PAM (Protospacer Adjacent Motif*)*. *Streptococcus pyogenes* (Sp) Cas9, which is the most used ortholog in genome editing, recognizes a 5’-NGG trinucleotide and its gene is 4.2 Kb coding for a 1333 amino acids (a.a.) protein [2]. Other Cas9s, such as *Neisseria meningitidis* Cas9 (NmCas9, 1082 a.a.), *Staphylococcus aureus* (SaCas9, 1053 a.a.) and the smalless one, *Campylobacter jejuni* Cas9 (CjCas9, 984 a.a.) bind respectively to 5’-NNNNGATT, 5’-NNGRRT and 5’NNNVRYM PAMs [3–6]. These Cas9 proteins that are smaller in size are thus more favorable for *in vivo* expression since its gene is more easily encapsulated with an adequate promoter and a sgRNA in a single AAV, the most important delivery system, which has a packaging limit of 4.7 kb [7]. AAV vectors produced with oversize genomes have lower particle number, lower transduction efficiency, and inefficient expression [8, 9]. Thus, the reduced sizes of these Cas9s, particularly CjCas9, make their vectorization easier. The CjCas9, the smaller one, permits its delivery with two sgRNAs by a single AAV particle [5].

Off-target problems are a concern for all CRISPR/Cas9 technologies, however, each Cas9 has a different PAM. A more complex PAM reduces the potential binding sites of the Cas9 and will thus decrease the probability of off-target mutations. We thus expect fewer potential off-target mutations using CjCas9, which the PAM correspond to 5’NNNVRYM3’, than with the SpCas9 that has a simple PAM (NGG) [10]. Indeed, to determine the genome-wide specificity of the CjCas9 nuclease, whole genome sequencing (Digenome-seq) was performed [11]. The authors reported that SpCas9 nucleases, designed to cleave sites that overlapped those of CjCas9, cleaved 105 off-target sites while CjCas9 cleaved only 8 off-target sites in the mouse genome [11].

However, CjCas9 activity seems to be less effective than SpCas9 [12] and may limit its use for clinical treatments. In this manuscript, we investigated whether this reduced activity is associated with the marked instability of the CjCas9 protein. We noted low amount of CjCas9 protein compared to SpCas9 protein in cells treated under the same conditions. It has been observed that Cas9 protein levels are directly correlated with genome edition efficiency [13]. Thus, we hypothesized that stabilizing the CjCas9 protein could enhance its nuclease activity.

Protein degradation is controlled by the ubiquitin (Ub)–proteasome system (UPS) and by autophagy–lysosome system [14]. Proteasome is a highly conserved multi-catalytic protease complex found in eukaryotes and present in both the nucleus and the cytoplasm. This complex cleaves and selectively degrades the peptide bonds of abnormal or short-lived proteins [15]. Several proteasome inhibitors able to block protein degradation pathway have been identified [16, 17]. Among them, proteasome inhibitor bortezomib has been accepted by the FDA as an anti-cancer medication [15, 18]. We have used it in our study to inhibit the degradation of CjCas9 protein in human cells. To confirm that CjCas9 preserves its activity with the treatment, we quantified the editing rate on the frataxin gene with and without a treatment with a proteasome inhibitor. Our results showed that the accumulation of this nuclease allowed more efficient editing on the *FXN* gene, i.e., a deletion of the GAA repeat. Our results provide evidence for the existence of a posttranslational regulation mechanism of CjCas9 levels mediated by the UPS and open the possibility to interfere with CjCas9 degradation to increase its stability in cells.

## Materials and methods

### Chemical reagents

The proteasome inhibitors MG132 and bortezomib were purchased from New England Biolab (NEB) and Sigma-Aldrich respectively. The purity of these inhibitors was >99%.

### Cell lines and transfection

HEK293T, HeLa, and Neuro-2a (N2A) (ATCC CCL-13I) cells were cultured in DMEM medium (DMEM, GIBCO-Thermo Fisher Scientific, Waltham, MA, USA) supplemented with 10% fetal bovine serum and 1% Penicillin/Streptomycin at 37°C with 5% CO_2_. Neuro-2a cells are mouse neuroblasts isolated from brain tissue. The SH-SY5Y (ATCC HTB-11) cells were cultured in DMEM/F12 with 10% fetal bovine serum and penicillin/streptomycin (1%) at 37°C with 5% CO_2._ The SH-SY5Y are human neuroblastoma cells.

The cells were transfected with Lipofectamine 2000 (Thermo Fisher Scientific Inc.) according to the manufacturer protocol. The cells were treated with different doses of the MG132 during 16 h starting 48 h after their transfection or with bortezomib during 48 h starting 24 h after transfection. Cells were collected 72 h after transfection. For editing assessment, bortezomib was administered 6 h before transfection with plasmid encoding CjCas9 and 2sgRNA targeting FXN intron 1 on both sides of GAAr. Endotoxin free plasmids were used for the transfection experiments.

### Plasmids and oligonucleotides

The pX551-CMV-SpCas9 (Addgene plasmid #107024) and the pX551-CMV-CjCas9 (Addgene plasmid #107035), with their Cas9 fused with an HA tag, were used to assess Cas9 expression with or without a proteasome inhibitor.

The plasmid (CjCas9/2sgRNA) coding for CjCas9 and included two U6-sgRNAs cassettes (expressing sgRNA-FXNU4 and sgRNA-FXND1) targeting the *FXN* intron 1 to remove the GAAr sequence. The sgRNAs were designed with the Benchling software (https://benchling.com) [5]. The human cytomegalovirus (CMV) promoter, the short form of the EF1α promoter (EFS) and the chicken β-actin promoter with the CMV enhancer (CBH) have been interchanged in the CjCas9/2sgRNA plasmid using restriction enzymes SpeI et AgeI.

### Western blots

Cells were transfected as described above, and protein lysates were prepared 72 hours post transfection. The protein concentrations were determined using an amido black assay. Protein samples were separated onto 4–15% Mini-PROTEAN® TGX Stain-Free™ Protein Gels (Biorad). Proteins were then transferred 1 hr onto a polyvinylidene fluoride membrane. That membrane was blocked for 2 h in 5% milk in 1X Tris-buffered saline (TBS) and 0.01% Tween 20 at room temperature. The CjCas9 and SpCas9 protein fused with HA-tag were detected using anti-HA tag (1:1,000; Cell Signaling Technology, Danvers, MA) and β-actin with anti-β-actin (1:1,000; Cell Signaling Technology) antibodies, respectively, and visualized with HRP conjugated anti-rabbit secondary antibody (1:20,000; Dianova, Hamburg, Germany) using West Pico Chemiluminescence substrate (Thermo Fisher Scientific, Waltham, MA). The revelation was made at different times of exposition to visualise all possible bands on the western.

### TIDE (Tracking of indels by decomposition)

Primers were designed to allow the amplification of 777 bp of the region targeted by two sgRNAs in *FXN* gene. PCR products were then Sanger sequenced, and the resulting files were analysed using the TIDE tool provided at http://shinyapps.datacurators.nl/tide/ [19] Amplifications of genomic DNA from untreated cells were used as control samples.

### Quantitative PCR

Genomic DNA was extracted from cells using the phenol:chloroform method. Plasmid copy numbers in harvested cells were determined by qPCR using SYBR Green master mix (Bio-Rad, Hercules, CA, USA) following the manufacturer’s instructions. F1 bacteriophage origin of replication sequence present in the plasmids pX551-CMV-SpCas9 and pX551-CMV-CjCas9 were amplified.

RT-qPCR was used to compare the mRNAs of CjCas9 and SpCas9. RNA was isolated with TRI reagent according to the manufacturer’s protocol (Sigma-Aldrich Inc., St-Louis, MO, USA). cDNA was then prepared using the high-capacity cDNA Reverse Transcription kits (Thermo Fisher Scientific Inc., MA, USA). The Bio-Rad C1000 Touch thermocycler was used for the measurement.

DdPCR was used to quantify the rate of frataxin gene editing. 100 ng of DNA and 1X ddPCR supermixes for probes no dUTP (deoxyuridine triphosphate) (Bio-Rad, Hercules, CA, USA) were used with two sets of primers/probes: reference primers/probes HEX (FXN wild type), and target primers/probes FAM (FXN edited) were used to generate the droplets using the QX200 droplet generator (Bio-Rad, Hercules, CA, USA). The droplets were transferred to a 96-well plate for a PCR of 95°C, 10 min (94°C, 30 s; 60°C, 60 s; 72°C, 30 s), 50 cycles, 98°C, 10 min. Ramp rate was adjusted to 2°/s at all steps. The 96-wells plate was then read by the QX200 Droplet reader (Bio-Rad, Hercules, CA, USA).

TIDE was used to assess the level of Indel induced by CjCas9 and the sgRNAs on the *FXN* gene amplified by PCR.

The sgRNA and the primer sequences are available in S1 and S2 Tables respectively.

### Statistical analysis

The results presented were analysed with Prism 9 (GraphPad Software, CA, USA). Specific statistical tests used, number of replicates and p values are included in the figure captions accompanying the data.

## Results

### CjCas9 protein stability compared to SpCas9 protein

**Fig 1:**
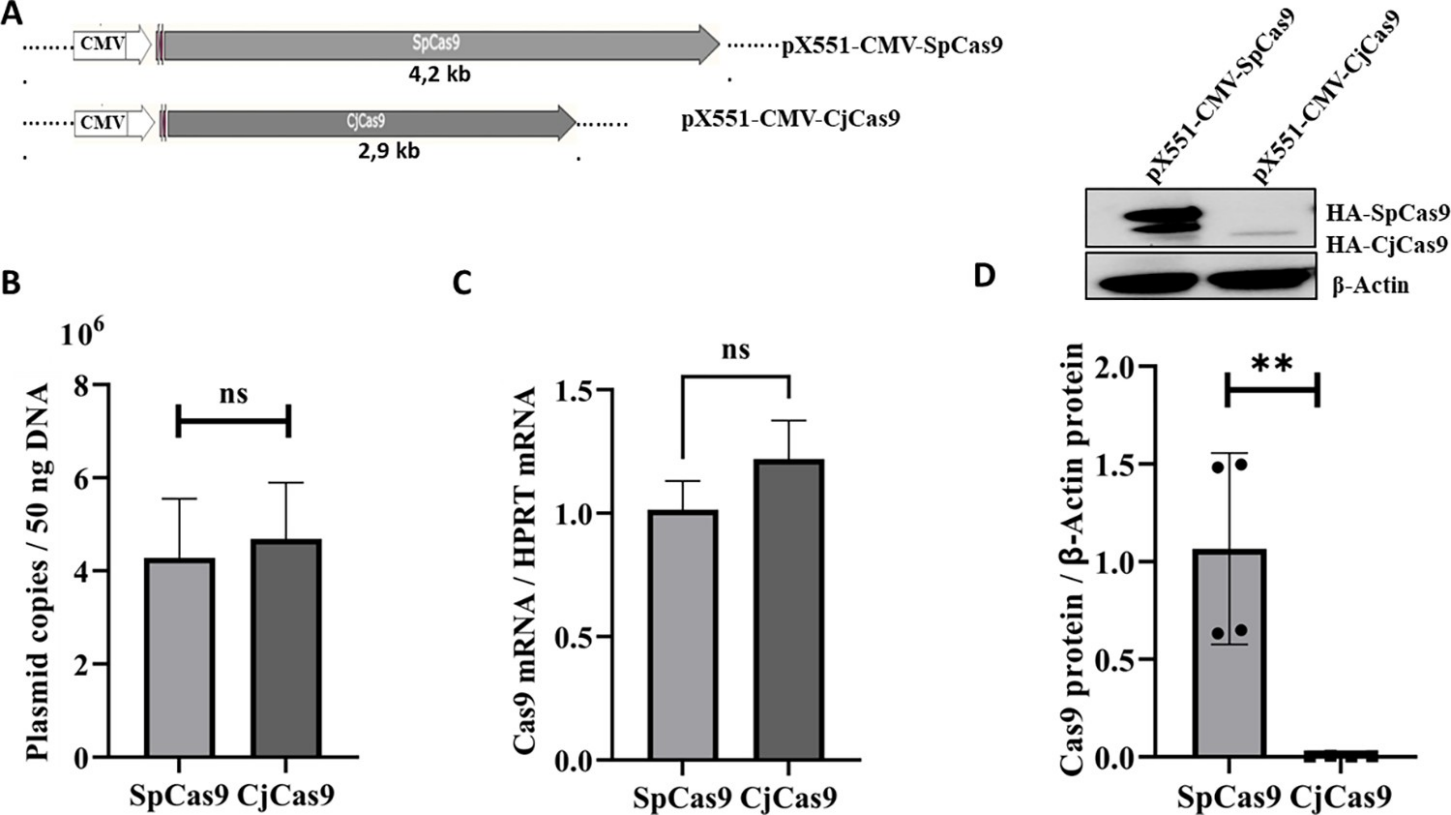
CjCas9 protein stability compared to SpCas9 protein. **A**) Schematic representation of the size of SpCas9 and CjCas9. **B)** The HEK293T cells were transfected with the same number of copies of plasmids of either pX551-CMV-SpCas9 or of pX551-CMV-CjCas9. 72 h later, the numbers of plasmids inside the cells were determined by qPCR. **C**) CjCas9 and SpCas9 mRNAs were assessed by RT-qPCR with HPRT as the reference gene. **D**) The concentrations of the two Cas9 proteins fused with HA-tag were evaluated by western blot. Data are means ± SEM (n ≥ 3), *p < 0.05, **p < 0.005, and ***p < 0.0005 (Student’s t tests).

We compared the stability of the CjCas9 protein with that of SpCas9. To do this, we transfected the same number of plasmids, i.e., 2.5.10^11^, of each gene under the same CMV promoter (pX551-CMV-SpCas9 #107024; pX551-CMV-CjCas9 #107035). The plasmid backbone (poly A, ori, AmpR…) is identical for both constructions. The CjCas9 gene being smaller than the SpCas9 gene, the pX551-CMV-CjCas9 plasmid is smaller than the pX551-CMV-SpCas9 plasmid (Fig 1A). This should favor the transfection of the former plasmid and a higher expression of CjCas9. 72 hours later, we analyzed DNA, RNA and proteins from treated HEK293T cells. The number of plasmid copies per nucleus and the mRNAs of the two nucleases were comparable, while the amounts of proteins evaluated by western blot were very different (Fig 1B and 1C). Indeed, CjCas9 accumulated very weakly. In this experiment, the CjCas9 protein concentration was less than 0.5% of the SpCas9 concentration (Fig 1D). The CjCas9 protein concentrations determined by western blot were very low in SH-SY5Y and N2A cells (S1 Fig).

### The proteasome inhibitor MG132 increased the CjCas9 protein level

**Fig 2:**
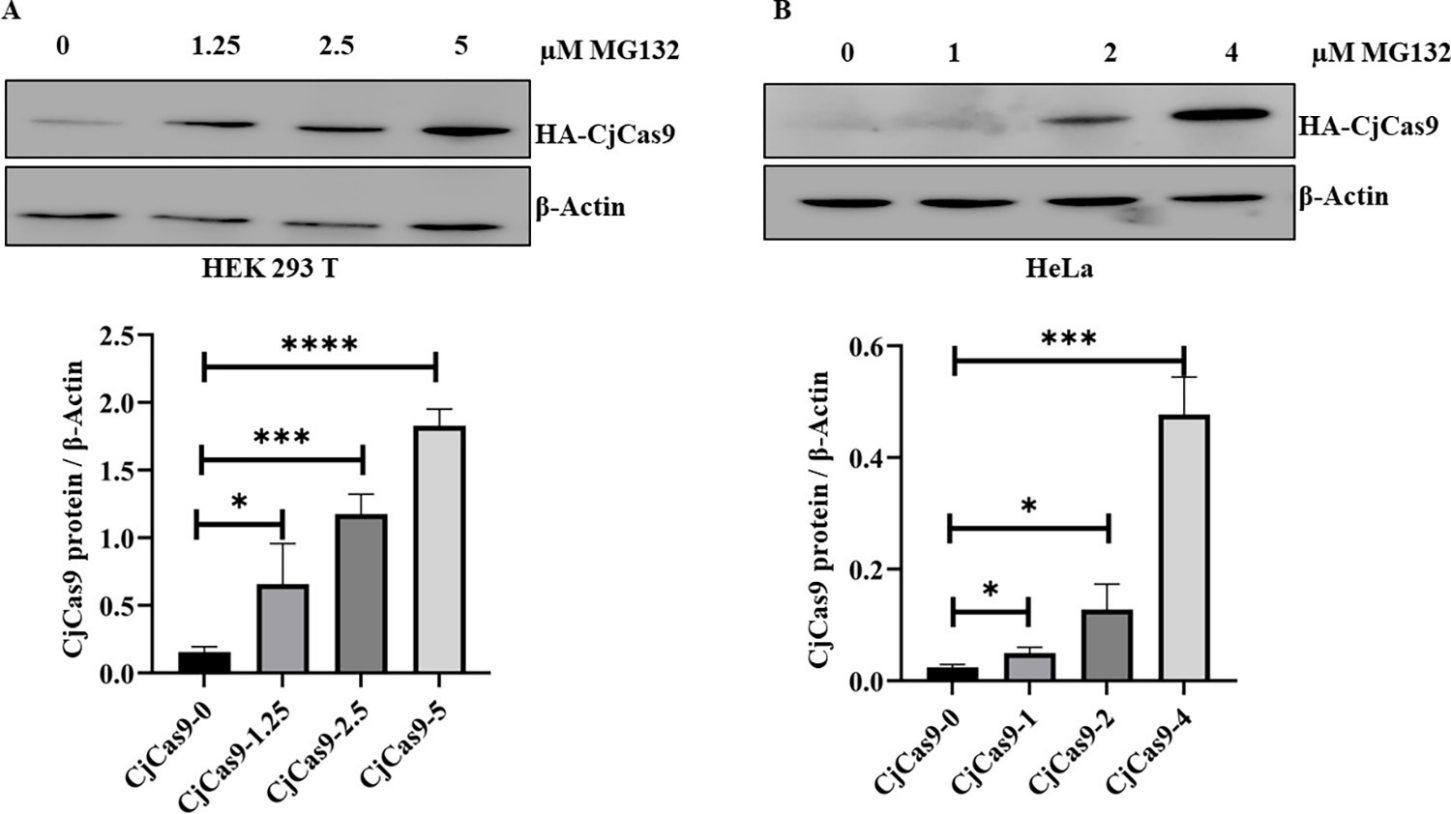
The proteasome inhibitor MG132 increases CjCas9 protein level. 48 hours after transfection of the cells (in **A** HEK293T cells and in **B** HeLa cells) with the pX551-CMV-CjCas9-HA plasmid, the wells were treated with different doses of the proteasome inhibitor MG132 (1 to 5 μM) for 16 hours. The proteins were analyzed 3 days post transfection by western blot with an anti-HA antibody. β-actin was used as a loading control. Data are means ± SEM (n ≥ 3), *p < 0.05, **p < 0.005, and ***p < 0.0005 (Student’s t tests).

We were interested to increase the transient accumulation of the CjCas9 protein within cells. Therefore, we verified whether the CjCas9 protein is regulated by the UPP degradative pathway. To address this question, we inhibited the proteasome in HEK293T cells and in HeLa cells, transfected with CjCas9 gene under CMV promoter to allow for increased protein accumulation. Fig 2 shows that cells treated with the proteasome inhibitor MG132 during 16 h accumulated significantly higher amounts of CjCas9 proteins compared with untreated cells. A dose-dependent accumulation of the CjCas9 protein was observed (Fig 2A and 2B). The level of CjCas9 proteins in the presence of the proteasome inhibitor MG132 approximated the protein level of SpCas9 proteins (S2 Fig). This experiment shows that the UPP was involved in regulating CjCas9 protein turnover and that the degradation of the CjCas9 can be blocked by MG132.

### Bortezomib reduced CjCas9 protein degradation

**Fig 3:**
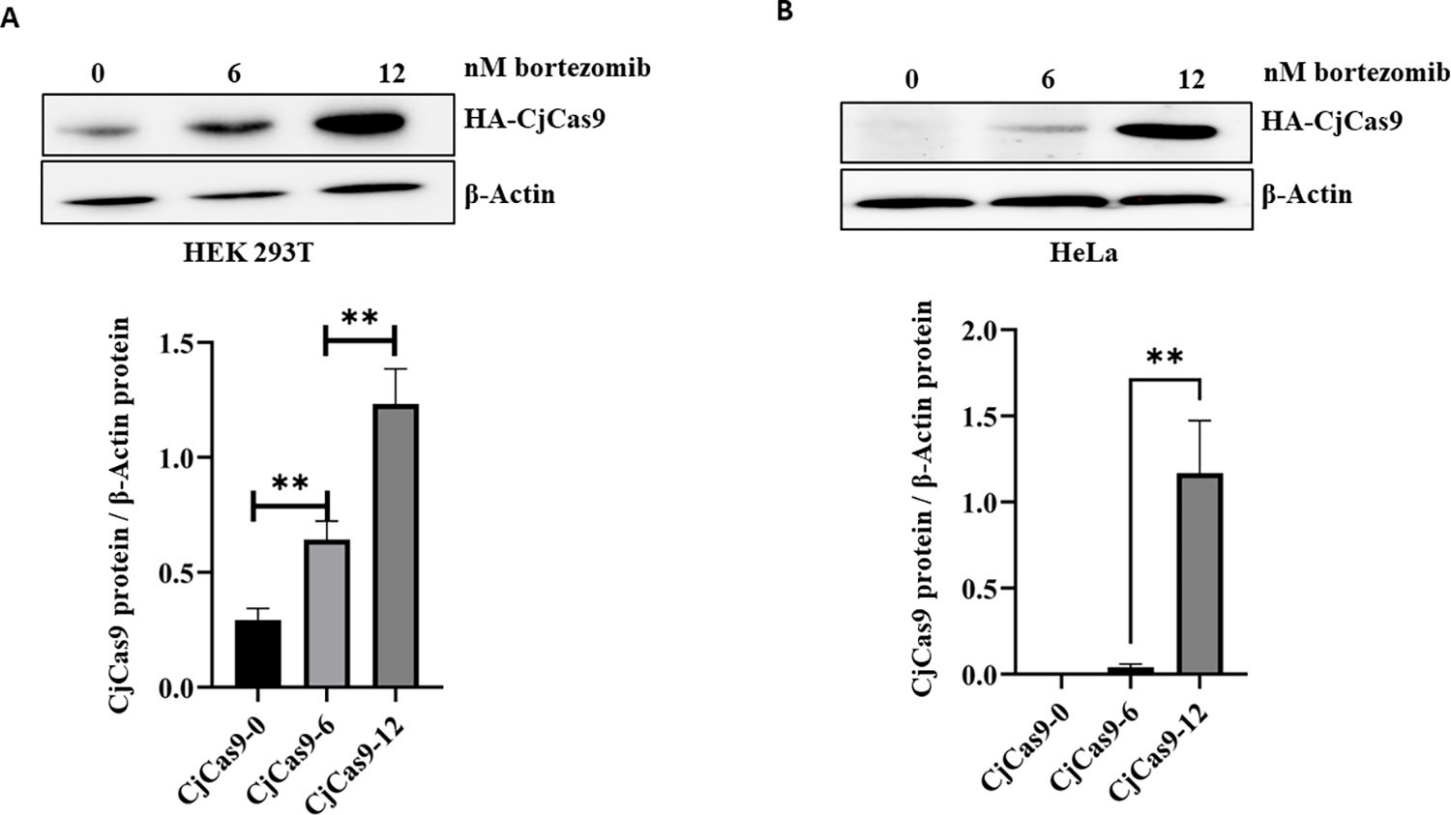
Proteasome inhibitor bortezomib reduces CjCas9 degradation. HEK293T cells (in **A**) and HeLa cells (in **B**) were transfected with equal concentrations of plasmids coding for HA-CjCas9 and treated with various concentrations of bortezomib (0, 6 and 12 nM) for 40 hours. Total cell extracts were analyzed by western blots with antibodies against an HA and β-actin (as a reference protein). Data are means ± SEM (n ≥ 3), *p < 0.05, **p < 0.005, and ***p < 0.0005 (Student’s t tests).

MG132 is not an FDA-approved drug, other FDA-approved proteasomal inhibitors such as bortezomib can also be used [15]. Therefore, we evaluated the effect of bortezomib on the accumulation of the CjCas9 proteins. This drug was accepted by the FDA in clinic and permits recovery of proteasome activity nearly 48–72 hours following bortezomib administration in preclinical models [18]. The cells were thus treated with different doses of bortezomib (6 and 12 nM) for 40 hours, starting 24 hours post-transfection with a plasmid encoding CjCas9 under a CMV promoter. After analysis of the proteins on western blots, we observed a dose-dependent accumulation of CjCas9 both in the HEK293T cells and in the HeLa (Fig 3A and 3B).

### Bortezomib improved the accumulation of CjCas9 under specific promoters weaker than the CMV promoter

**Fig 4:**
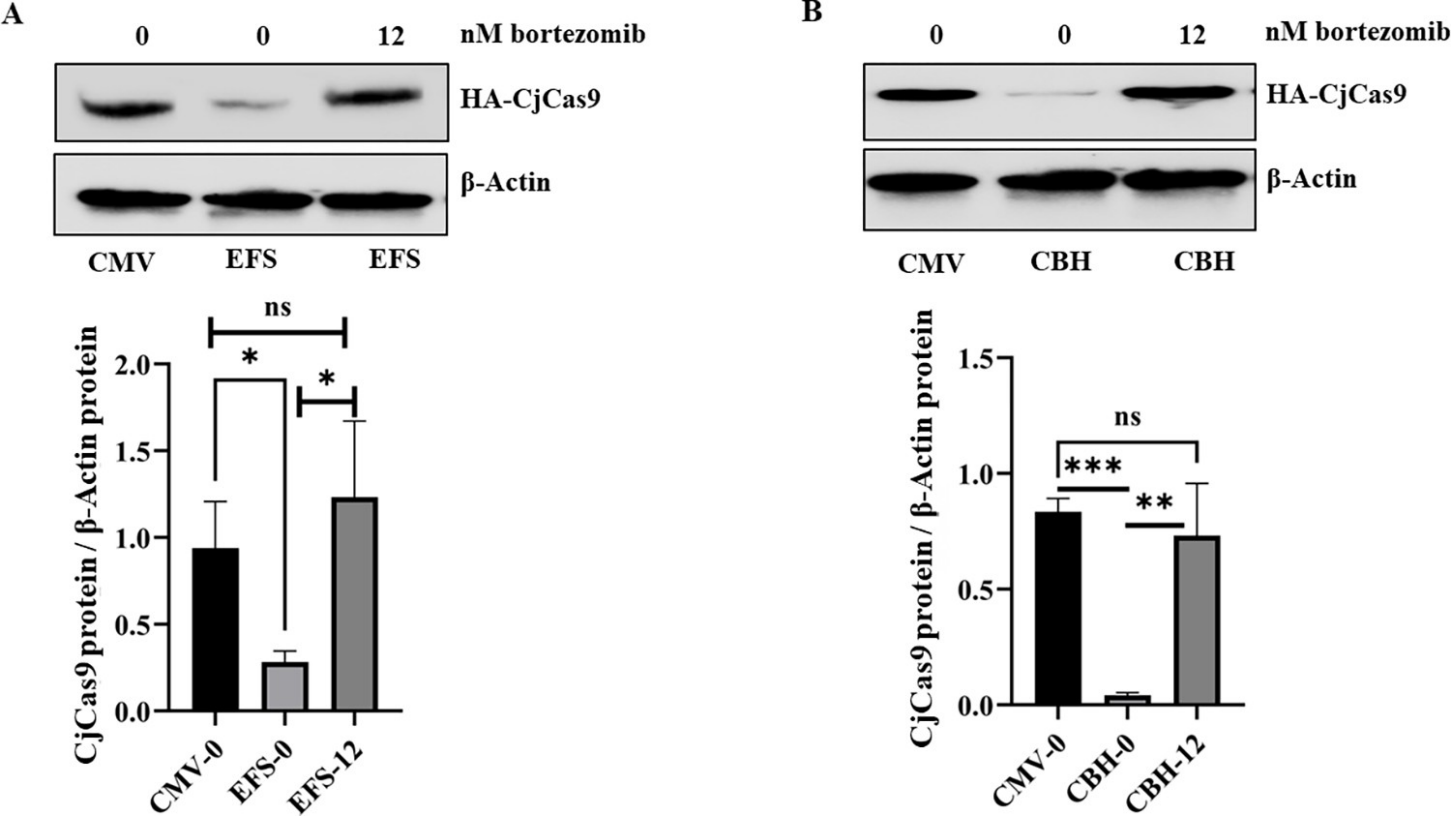
Bortezomib increases the stability of CjCas9 synthesized under a weaker promoter than CMV. The same number of plasmids coding for CjCas9-HA under different promoters, i.e., **A**) EFS or **B**) CBH, were transfected in HEK293T cells. Some of the wells was treated 18 hours after transfection with 12 nM of bortezomib for 40 hours. The cells were harvested and the level of the HA-CjCas9 protein was evaluated by western blots with β-actin as the reference protein. Untreated wells and CjCas9 under CMV promoter served as control. Data are mean ± SEM (n ≥ 3), *p < 0.05, **p < 0.005, and ***p < 0.0005 (Student’s t test).

Increasingly, the Cas9 and sgRNA sequences are modified to improve the efficiency of the CRISPR system. These sequence modifications include peptides interacting with chromatin or sequences allowing to control the expression of Cas9 [20–22]. The use of smaller promoters would be an asset to meet the challenge of using a single AAV whose encapsulation limit is 4.7 kb. However, the use of certain more specific but weaker promoters may benefit from the transient inhibition of the proteasome. Therefore, the CjCas9 protein expressed under the CMV promoter (508 bp) was compared with its expression under less powerful promoters such as EFS (212 bp) or CBH (794 bp) with or without a bortezomib treatment. After a 40 hours treatment of HEK293T cells with 12 nM of bortezomib, a noticeable accumulation of CjCas9 protein comparable to the expression under CMV was seen (Fig 4A and 4B). In a similar experiment, an increase in the CjCas9 protein under the MiniCMV (39 bp) promoter was also noted, but the CjCas9 protein concentration was well below the concentration observed with the CMV promoter (S3 Fig). Thus, it is possible to express the CjCas9 protein under small promoters, however they still must be effective enough to reach a level of CjCas9 protein sufficient to eventually permit a good level of gene editing.

### Bortezomib enhanced CjCas9 nuclease activity in HEK293T cells

**Fig 5:**
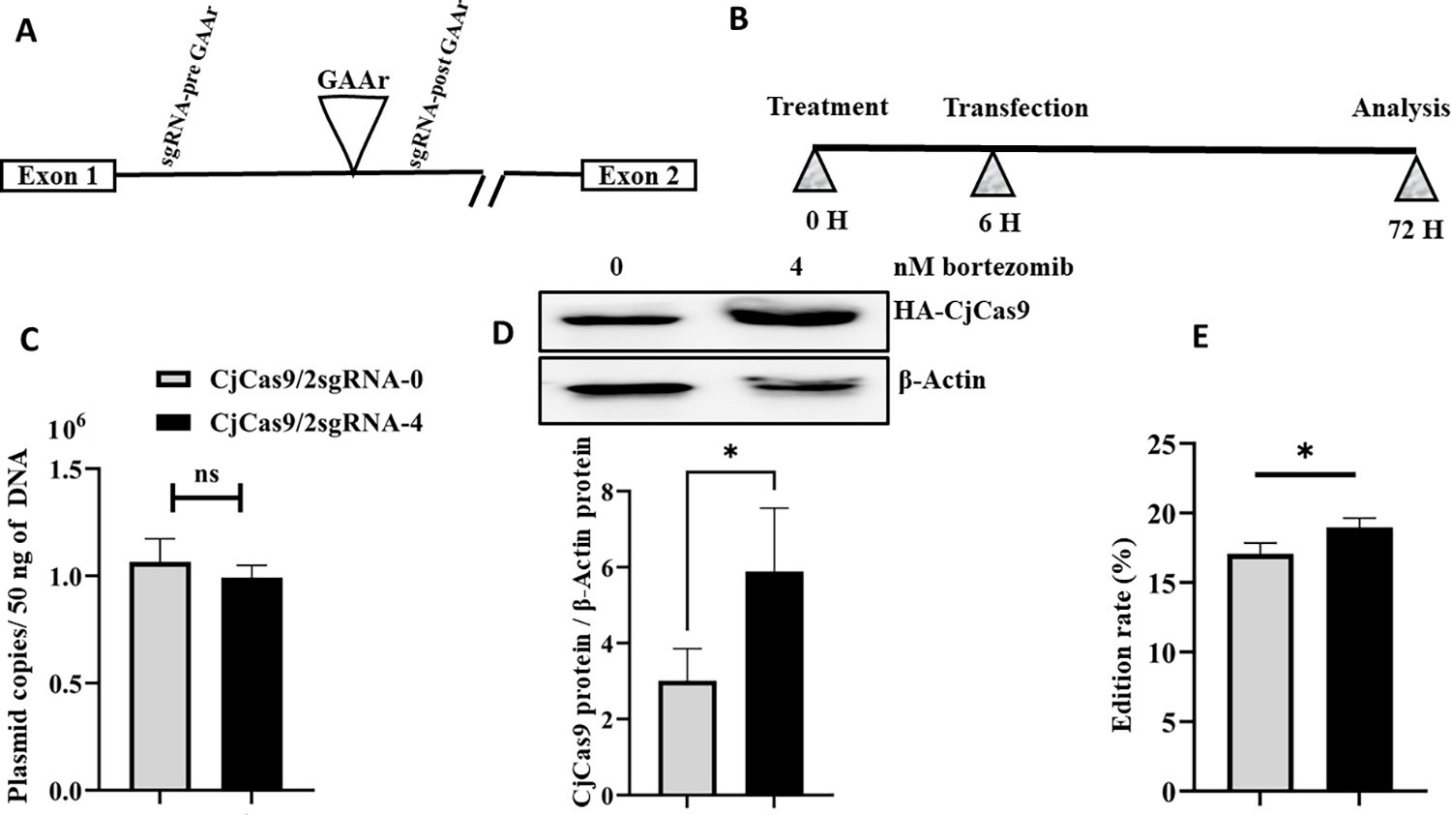
Bortezomib enhances the efficiency of CjCas9/ 2 sgRNA-mediated GAA repeat deletion in the *FXN* gene of HEK293T cells. **A**) Schematic representation of the human *FXN* gene with the positions of the sgRNAs used in our experiment. **B**) Schematic description of the experiment: cells were co-treated with or without bortezomib (4 nM) and a plasmid encoding CjCas9 under CMV promoter and 2 sgRNAs under U6 promoters. **C**) The number of plasmid copies was determined by qPCR. **D**) The level of CjCas9 protein was quantified by western blot and normalized with β-actin. **E**) 72 hours after transfection, gene editing efficiency on *FXN* gene was measured by ddPCR. Data are means ± SEM (n ≥ 3), *p < 0.05, **p < 0.005, and ***p < 0.0005 (Student’s t tests).

Cas9 nuclease activity is known to increase with higher levels of Cas9 protein. We checked whether the accumulation of the CjCas9 proteins caused by bortezomib led to an increase in its efficacy on human *FXN* gene in HEK293T cells. Our desire to have the bortezomib drug present throughout the edition (therefore 72 h), led us to reduce its concentration to avoid cellular toxicity. Therefore, 4 nM of bortezomib was added to the culture medium starting 6 h before the transfection of a plasmid encoding CjCas9 under the CMV promoter and 2 sgRNAs targeting a region before and after the GAAr in FXN intron 1. The medium was replaced by fresh medium also containing the drug 24 h and 48 h after the transfection (Fig 5A and 5B). The effectiveness of the edition was evaluated by ddPCR with and without bortezomib treatment. Analysis of the results showed an increase of the editing level (i.e., deletion of the GAAr) in the presence of the bortezomib without an impact on the transduction (Fig 5C and 5E). An increase in CjCas9 protein was observed following the treatment with 4 nM of bortezomib for 72 hours (Fig 5D). Evaluation of the indel rate induced by the 2 guides targeting sequences upstream of the GAAr (sgRNA-M1, sgRNA-M2) showed an increase in the presence of bortezomib (S4 Fig). Therefore, a temporarily increase of CjCas9 protein concentration is a good approach to increase the editing rate of the *FXN* gene and probably of other genes.

## Discussion

Small molecules are used in many approaches to improve transgene expression [23]. Indeed, strategies using molecules to control the activity of the CRISPR system, improved the system’s accessibility to heterochromatin and increased the transduction of cells by AAV vectors have been explored [5, 24–27]. It seems interesting that these molecules are useful to meet the challenges that the CRISPR system is facing. These molecules can be temporally regulated to inhibit or activate specific targeting processes, and these effects are often reversible [25]. In the present manuscript, we showed a low stability of the CjCas9 protein compared to the SpCas9 protein. The reason for this instability is not known. It is possible that this reduced stability is due to unfolding or misfolding of the CjCas9 protein. Selective inhibition of the UPR (Unfolded Protein Response) mechanism could help to identify the cause of this instability [28].

The small size of the CjCas9 is a great advantage to overcome the packaging size limitation of AAVs. Its complex PAM and its sgRNA length (22-nt) are also advantageous since they reduce off target mutations compared to the SpCas9 [6, 12, 29]. Our experiments demonstrated that a transient proteasome inhibition is as a strategy to enhance CjCas9 accumulation. This indicates that the CjCas9 rapid degradation was mediated by the ubiquitin-dependent pathway, i.e., by the proteasome. Since the activity of the proteasome is variable in different cell types [30], the activity of CjCas9 is also tissue dependent. For example, low CjCas9 concentrations were observed in N2A and in SH-SY5Y neuroblastomas. This implies that the use of CjCas9 *in vivo* first requires tests to determine the protein concentration which is obtainable in the specific targeted cells.

The accumulation of CjCas9 protein trigger by proteasome inhibition improved editing of the *FXN* gene in human cells. Even if this increase was low *in vitro*, it may be an important factor *in vivo* where the transduction rate is very low. Indeed, a significant amount of plasmid copies accumulates in cells following in *vitro* transfections [31], however, such high DNA quantities are usually not obtained *in vivo* (less than 1 copy of AAV per nucleus in heart with AAV9) [32]. Therefore, the inhibition of Cas9 degradation in cells harboring one virus copy is important to increase the level of Cas9 proteins to achieve a better editing. This strategy can be useful *in vivo* to increase the efficiency of editing by the CRISPR system. Using only a transient administration of bortezomib may be ideal to obtain low off-target mutations. In addition, the use of proteasome inhibitors may be interesting to improve AAV transduction [33, 34]. However, a sustained inhibition of the proteasome activity can have harmful consequences for the cells because malfunctioning proteins can lead to cancer or neurological disorders [15, 35]. Therefore, it is important to degrade abnormal proteins as quickly as possible for the cells to survive. This is why, we suggest a strategy which consists of only a transient treatment with a proteasome inhibitor only during the editing period with a molecule whose degradation will restore the proteasome activity [36]. Food and Drug Administration (FDA) approved proteasome inhibitors, such as borteozomib, carfilzomib and Ixazomib, can be valuable for *in vivo* targeted gene editing and therapeutic research [36–38]. Ixazomib dissociates more rapidly from the proteasome than bortezomib, consistent with faster recovery of proteasome activity observed in the Proteasome-Glo assay. Interesting, several next-generation proteasome inhibitors are currently being investigated [39].

Our results thus provide the evidence for lower stability of CjCas9 and open the possibility to interfere with CjCas9 protein degradation to increase its stability for gene editing. A more targeted approach would be to determine the residue responsible for CjCas9 protein ubiquitination and degradation to have a more specific treatment. Either a modification of the amino acid in question or the use of small molecules designed to directly target this residue to prevent ubiquitin-dependent degradation [40, 41]. Furthermore, since proteasome inhibitors can increase frataxin stability [17] and improve AAV transduction [33], it is expected to have a more positive effect on the therapy of Friedreich ataxia by GAA repeat deletion in transgenic animals.

Our studies support, the use of adapted and temporary molecules to improve the performance of the CRISPR/Cas9 system.

## Supporting information

S1 Fig Stability of CjCas9 protein in human and mouse neuroblastomas. The same number of copies of plasmids encoding SpCas9 (pX551-CMV-SpCas9) and CjCas9 (pX551-CMV-CjCas9) were transfected in the HEK293T cells and in the **A**) N2A and **B**) SH−SY5Y cells. 72 hours after, HA-SpCas9 and HA-CjCas9 proteins were revealed by western blot with HA antibody. β- actin was used as a reference protein.
(PDF)

S2 Fig Comparison between the level of the SpCas9 protein and that of CjCas9 in the presence of MG132. HEK 293T cells were transfected with equal copies of plasmids encoding HA-SpCas9 or HA-CjCas9. Wells of CjCas9 were treated with various concentrations of MG132 for 16 hours. The HA-SpCas9 and HA-CjCas9 protein levels were determined by Western blot with a HA antibody. β-actin was used as an internal control. Data are means ± SEM (n ≥ 3), *p < 0.05, **p < 0.005, and ***p < 0.0005 (Student’s t tests).
(PDF)

S3 Fig Bortezomib increase the level of CjCas9 synthesized under the mini-CMV promoter. HEK 293T were treated with the same number of copies of plasmid encoding CjCas9 under Mini-CMV and CMV promoter. The wells with Mini-CMV were treated with 12 nM of bortezomib. The CjCas9 protein fused with HA was revealed by western blot. Data are means ± SEM (n ≥ 3), *p < 0.05, **p < 0.005, and ***p < 0.0005 (Student’s t tests).
(PDF)

S4 Fig Bortezomib enhances the efficiency of the CRISPR/Cas9-mediated deletion in the *FXN* gene of HEK293T cells. A) Diagrams of the localization of sgRNAs and primers used in this experiment. B) 6 hours before the transfection the medium was renewed with medium containing 0 or 4 nM of bortezomib. A plasmid encoding CjCas9 and a specific sgRNA (M1 or M2) was transfected into HEK 293T cells. The cells were harvested 72 hours after treatment. The genomic DNA PCR product was sequenced by Sanger sequencing and the TIDE software was used to determine the indel level. Data are means ± SEM (n ≥ 3), *p < 0.05, **p < 0.005, and ***p < 0.0005 (Student’s t tests).
(PDF)

S1 Table sgRNA used in this study. (PDF)

S2 Table Primers used in this study. (PDF)
