# Supplementary material for: Small-molecule inhibitors of proteasome increase CjCas9 protein stability
Source: PLoS One. 2023 Jan 19;18(1):e0280353. doi: 10.1371/journal.pone.0280353 (PMC9851528; doi:10.1371/journal.pone.0280353)
Supplement: S1 Fig — The same number of copies of plasmids encoding SpCas9 (pX551-CMV-SpCas9) and CjCas9 (pX551-CMV-CjCas9) were transfected in the HEK293T cells and in the A) N2A and B) SH−SY5Y cells. 72 hours after, HA-SpCas9 and HA-CjCas9 proteins were revealed by western blot with HA antibody. β- actin was used as a reference protein. (PDF) [file pone.0280353.s001.pdf]

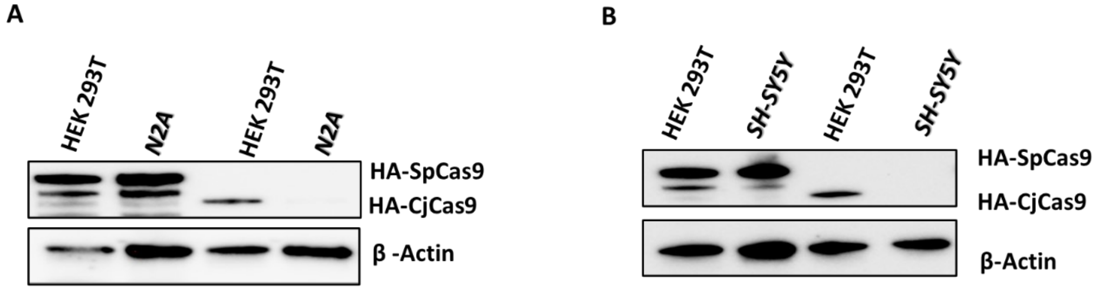

S1 Fig: Stability of CjCas9 protein in human and mouse neuroblastomas. The same number of copies of plasmids encoding SpCas9 (pX551-CMV-SpCas9) and CjCas9 (pX551-CMV-CjCas9) under the CMV promoter were transfected onto the HEK293T cells and onto the **A**) N2A and **B**) SH−SY5Y cells. 72 hours after, HA-SpCas9 and HA-CjCas9 proteins were revealed by western blot with HA antibody. β- actin was used as a reference protein**.**
