# Supplementary material for: Small-molecule inhibitors of proteasome increase CjCas9 protein stability
Source: PLoS One. 2023 Jan 19;18(1):e0280353. doi: 10.1371/journal.pone.0280353 (PMC9851528; doi:10.1371/journal.pone.0280353)
Supplement: S2 Fig — HEK 293T cells were transfected with equal copies of plasmids encoding HA-SpCas9 or HA-CjCas9. Wells of CjCas9 were treated with various concentrations of MG132 for 16 hours. The HA-SpCas9 and HA-CjCas9 protein levels were determined by Western blot with a HA antibody. β-actin was used as an internal control. Data are means ± SEM (n ≥ 3), *p < 0.05, **p < 0.005, and ***p < 0.0005 (Student’s t tests). (PDF) [file pone.0280353.s002.pdf]

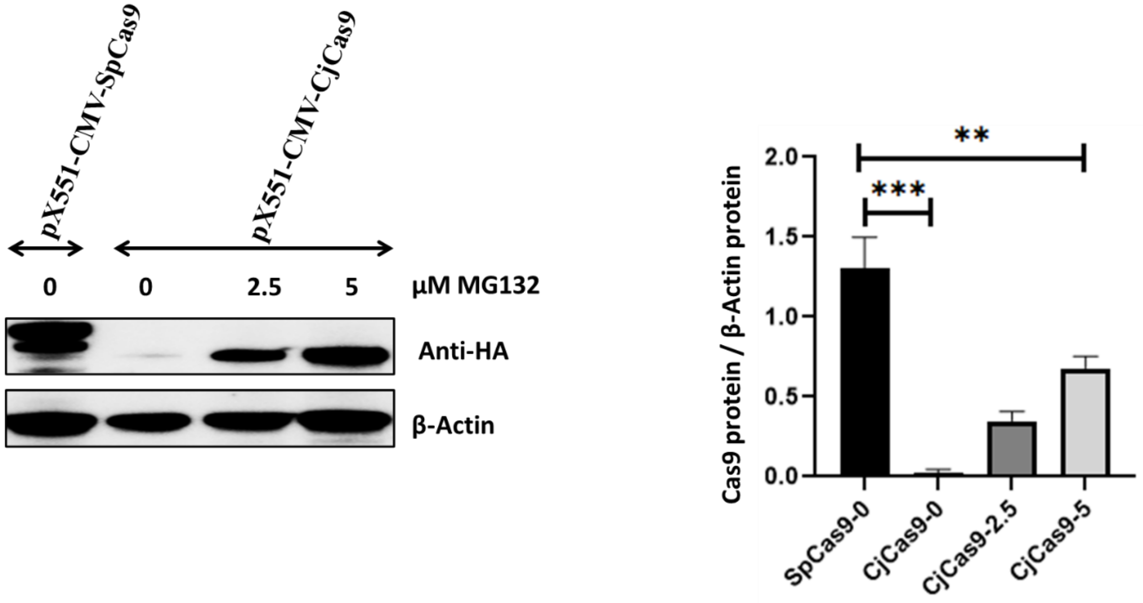

S2 Fig: Comparison between the level of the SpCas9 protein and that of CjCas9 in the presence of MG132. HEK 293T cells were transfected with equal copies of plasmid encoding for SpCas9 and CjCas9. Wells of CjCas9 were treated with various concentrations of MG132 for 16 hours. The HA-SpCas9 and HA-CjCas9 levels were determined by Western blot with HA antibody. b-actin was used as an internal control. Data are mean ± SEM ($n \geq 3$), *$p < 0.05$, **$p < 0.005$, and ***$p < 0.0005$ (Student's t test).
