# Supplementary material for: Small-molecule inhibitors of proteasome increase CjCas9 protein stability
Source: PLoS One. 2023 Jan 19;18(1):e0280353. doi: 10.1371/journal.pone.0280353 (PMC9851528; doi:10.1371/journal.pone.0280353)
Supplement: S3 Fig — HEK 293T were treated with the same number of copies of plasmid encoding CjCas9 under Mini-CMV and CMV promoter. The wells with Mini-CMV were treated with 12 nM of bortezomib. The CjCas9 protein fused with HA was revealed by western blot. Data are means ± SEM (n ≥ 3), *p < 0.05, **p < 0.005, and ***p < 0.0005 (Student’s t tests). (PDF) [file pone.0280353.s003.pdf]

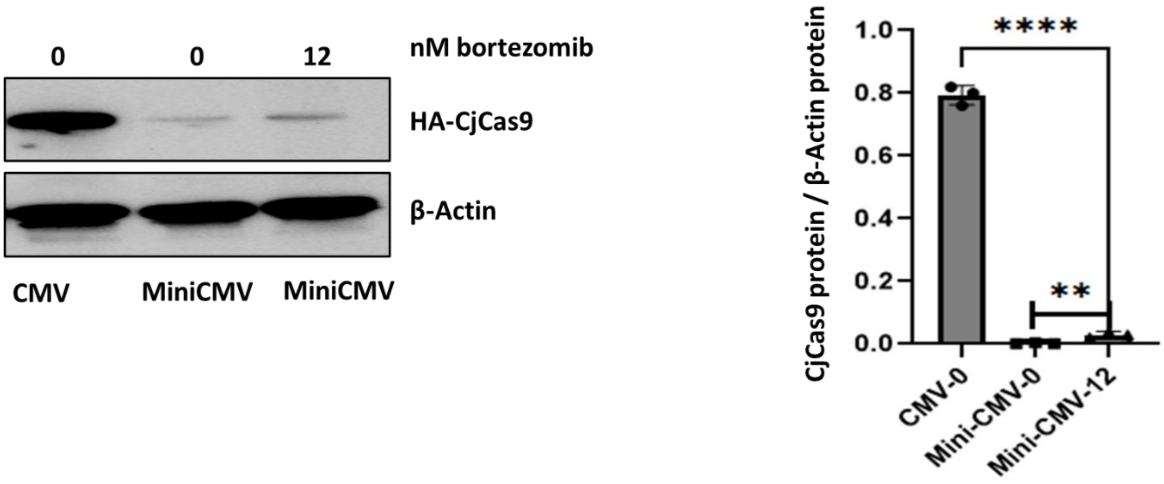

S3 Fig: Bortezomib increase the level of CjCas9 synthesized under the mini-CMV promoter. HEK 293T were treated with the same copies of plasmid encoding CjCas9 under Mini-CMV and CMV promoter. The wells with Mini-CMV were treated with 12 nM of bortezomib. The CjCas9 protein fused with HA was revealed by western blot. Data are mean ± SEM ($n \geq 3$), *$p < 0.05$, **$p < 0.005$, and ***$p < 0.0005$ (Student's t test).
