# Supplementary material for: Small-molecule inhibitors of proteasome increase CjCas9 protein stability
Source: PLoS One. 2023 Jan 19;18(1):e0280353. doi: 10.1371/journal.pone.0280353 (PMC9851528; doi:10.1371/journal.pone.0280353)
Supplement: S4 Fig — A) Diagrams of the localization of sgRNAs and primers used in this experiment. B) 6 hours before the transfection the medium was renewed with medium containing 0 or 4 nM of bortezomib. A plasmid encoding CjCas9 and a specific sgRNA (M1 or M2) was transfected into HEK 293T cells. The cells were harvested 72 hours after treatment. The genomic DNA PCR product was sequenced by Sanger sequencing and the TIDE software was used to determine the indel level. Data are means ± SEM (n ≥ 3), *p < 0.05, **p < 0.005, and ***p < 0.0005 (Student’s t tests). (PDF) [file pone.0280353.s004.pdf]

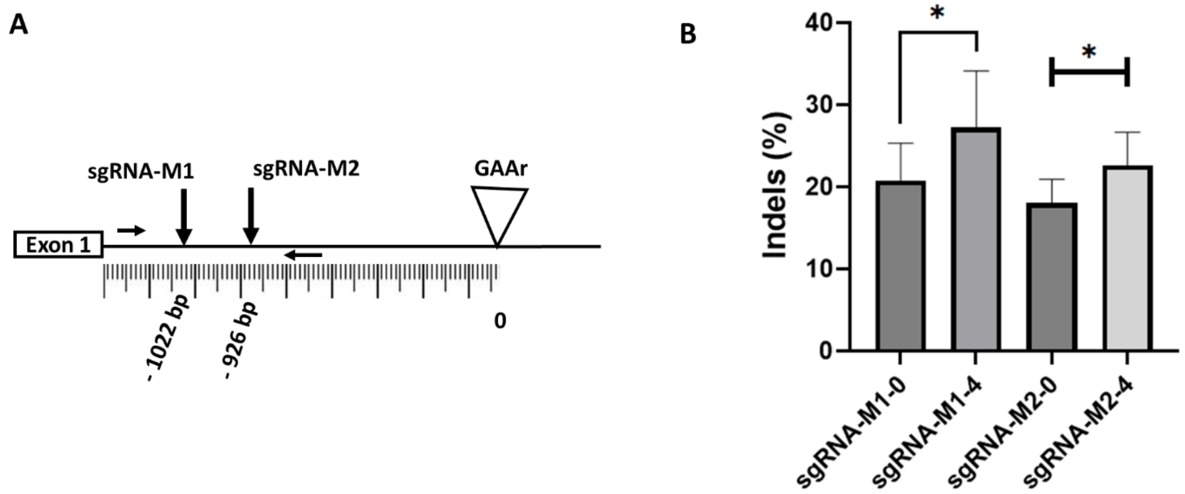

S4 Fig: **Bortezomib enhances CRISPR/Cas9-mediated knock-in efficiency on *FXN* gene in HEK293T cells** A) Diagrams of the localization of sgRNA and primers used in this experiment. B) 6 hours before the transfection the medium was renewed with medium containing 0 or 4 nM of bortezomib. A plasmid encoding CjCas9 and a specific sgRNA (M1 or M2) was transfected onto HEK 293T cells. 72 hours after treatment the cells were harvested. The genomic DNA PCR product was sequenced by Sanger sequencing and the TIDE software was used to determine the indel level. Data are mean ± SEM ($n \geq 3$), *$p < 0.05$, **$p < 0.005$, and ***$p < 0.0005$ (Student's t test).
