# Supplementary material for: Small-molecule inhibitors of proteasome increase CjCas9 protein stability
Source: PLoS One. 2023 Jan 19;18(1):e0280353. doi: 10.1371/journal.pone.0280353 (PMC9851528; doi:10.1371/journal.pone.0280353)
Supplement: S1 Table — (PDF) [file pone.0280353.s005.pdf]

S1 Table: sgRNA used in this study.

**Cas9**	**Name**	**Target strand**	**Sequence (5' – 3')**
**CjCas9**			
	sgRNA-FXNU4	**Anti-sens**	CCCGTGCGCGCAAACACACACA
	sgRNA-FXND1	**Anti-sens**	GAGGAGATCTAAGGACCATCAT
	M1	**Anti-sens**	GAAGTGACAAGCATGGAGACAG
	M2	**Anti-sens**	CACGCTATATACAGATACACAC
