# Supplementary material for: Small-molecule inhibitors of proteasome increase CjCas9 protein stability
Source: PLoS One. 2023 Jan 19;18(1):e0280353. doi: 10.1371/journal.pone.0280353 (PMC9851528; doi:10.1371/journal.pone.0280353)
Supplement: S2 Table — (PDF) [file pone.0280353.s006.pdf]

S2 Table: Primers used in this study.

**Method**<br>Name	**Orientation**	**Sequence (5' – 3')**
**PCR**<br>Amplifies the region of intron 1 of the *FXN* gene targeted by the sgRNAs: M1 and M2		
Primer A F	Forward	TGGTTCTCCCGGTTGCATTT
Primer A R	Reverse	CCTCGTGAAACACCCTCTACC
**qPCR**<br>Amplifies f1 bacteriophage origin of replication sequence present in the plasmids (pX551-CMV-SpCas9 and pX551-CMV-CjCas9)		
Primer P F	Forward	TTCCCTTCCTTTCTCGCCAC
Primer P R	Reverse	GCCCACTACGTGAACCATCA
**RTqPCR**<br>Amplifies CjCas9 sequences		
Primer CjCas9 F	Forward	CGAGAAGGAGGTCATCGCAAAG
Primer CjCas9 R	Reverse	GTTACTTCACCGAGTGCGGAC
**RTqPCR**<br>Amplifies SpCas9 sequences		
Primer SpCas9 F	Forward	AACCTATGCCCACCTGTTCG
Primer SpCas9 R	Reverse	AGGATTGTCTTGCCGGACTG
**ddPCR**		
FXN wild type<br>Amplifies a sequence of intron 1 of the *FXN* gene not affected by the editing		
Forward	GGTTGCATTTACACTGGCTTC	
Reverse	AGAGAAGTGACAAGCATGGAG	
Probe	AGTCGCACCGCAGGACAAAATG	
FXN edited<br>Amplifies only edited alleles		
Forward	GTGTGTGTCTGTGTGTATCTGTAT	
Reverse	CCAATACGTGGCAGCTCAG	
Probe	TGCACAGAAGCCAAGTAATAAATGTCTGC	
